# Supplementary material for: Glial Draper signaling triggers cross-neuron plasticity in bystander neurons after neuronal cell death in Drosophila
Source: Nat Commun. 2023 Jul 24;14:4452. doi: 10.1038/s41467-023-40142-y (PMC10366216; doi:10.1038/s41467-023-40142-y)
Supplement: Supplementary file 1 — Supplementary information [file 41467_2023_40142_MOESM1_ESM.pdf]

Supplementary Figures

Wang et al., 2023

Nature Communications

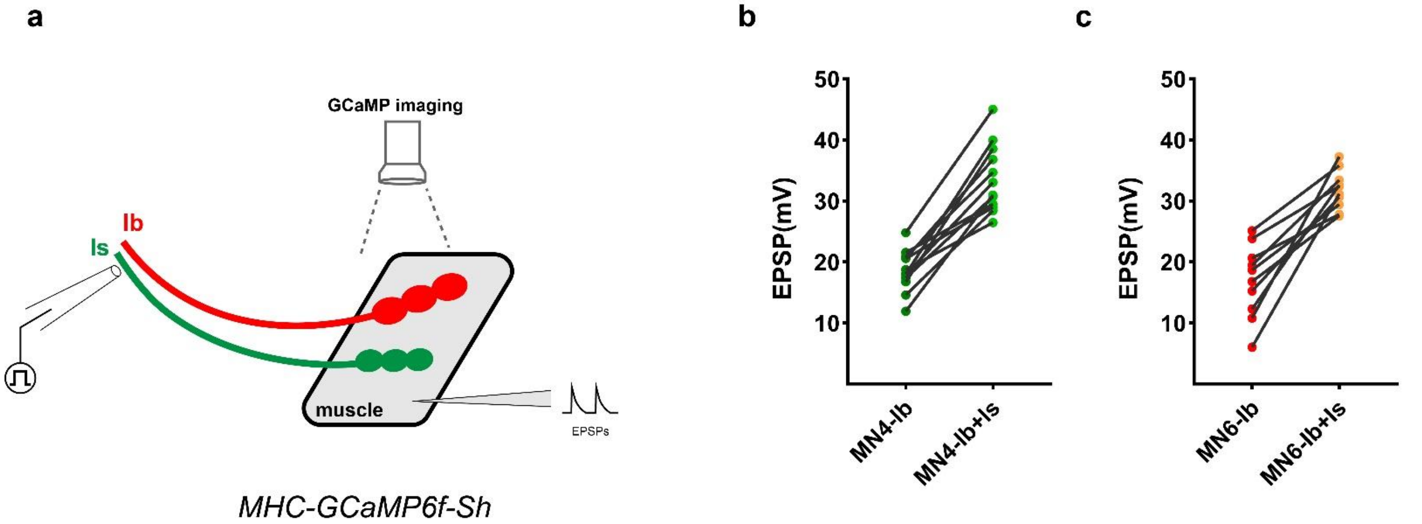

	EPSP (mV)
MN4-Ib	18.66±0.98
MN4-Ib+Is	33.60±1.60
Ib/Ib+Is	0.56±0.03

	EPSP (mV)
MN6-Ib	16.91±1.79
MN6-Ib+Is	31.82±0.96
Ib/Ib+Is	0.53±0.05

Supplementary Fig. 1. GCaMP imaging combined with electrophysiology recording to separate Ib and Is MN activity.

a. Schematic of the experimental setup. A GCaMP movie was recorded while recording EPSPs.

b. Paired MN4-Ib and MN4-Ib+Is EPSP amplitudes. N (NMJs) = 12 and 12.

c. Paired MN6-Ib and MN6-Ib+Is EPSP amplitudes. N (NMJs) = 10 and 10.

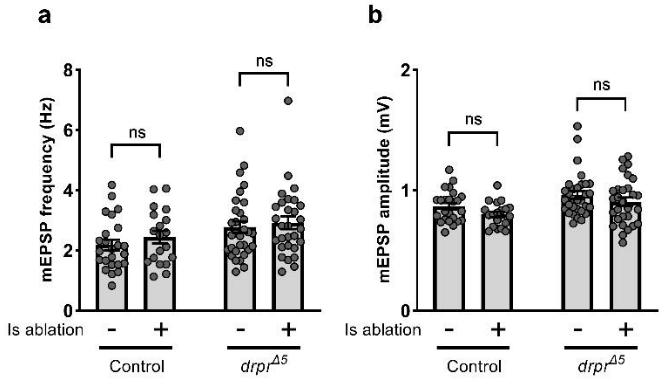

Supplementary Fig. 2. Quantification of mEPSPs of non-ablated and Is ablated larvae in control and *drpr*$^{\Delta 5}$ backgrounds.

a. Quantification of mEPSP frequencies in non-ablated and Is ablated larvae in control and *drpr*$^{\Delta 5}$ backgrounds. Control, t(41)=0.9502, p=0.3476, unpaired t-test, two-tailed. *drpr*$^{\Delta 5}$, t(58)=0.5102, p=0.6118, unpaired t-test, two-tailed.

b. Quantification of mEPSP amplitude in non-ablated and Is ablated larvae in control and *drpr*$^{\Delta 5}$ backgrounds. Control, t(41)=1.880, p=0.0672, unpaired t-test, two-tailed. *drpr*$^{\Delta 5}$, t(58)=1.169, p= 0.2471, unpaired t-test, two-tailed. For a and b, N (NMJs) = 24, 19, 31, 29.

Error bars indicate ± SEM, ns = non-significant.

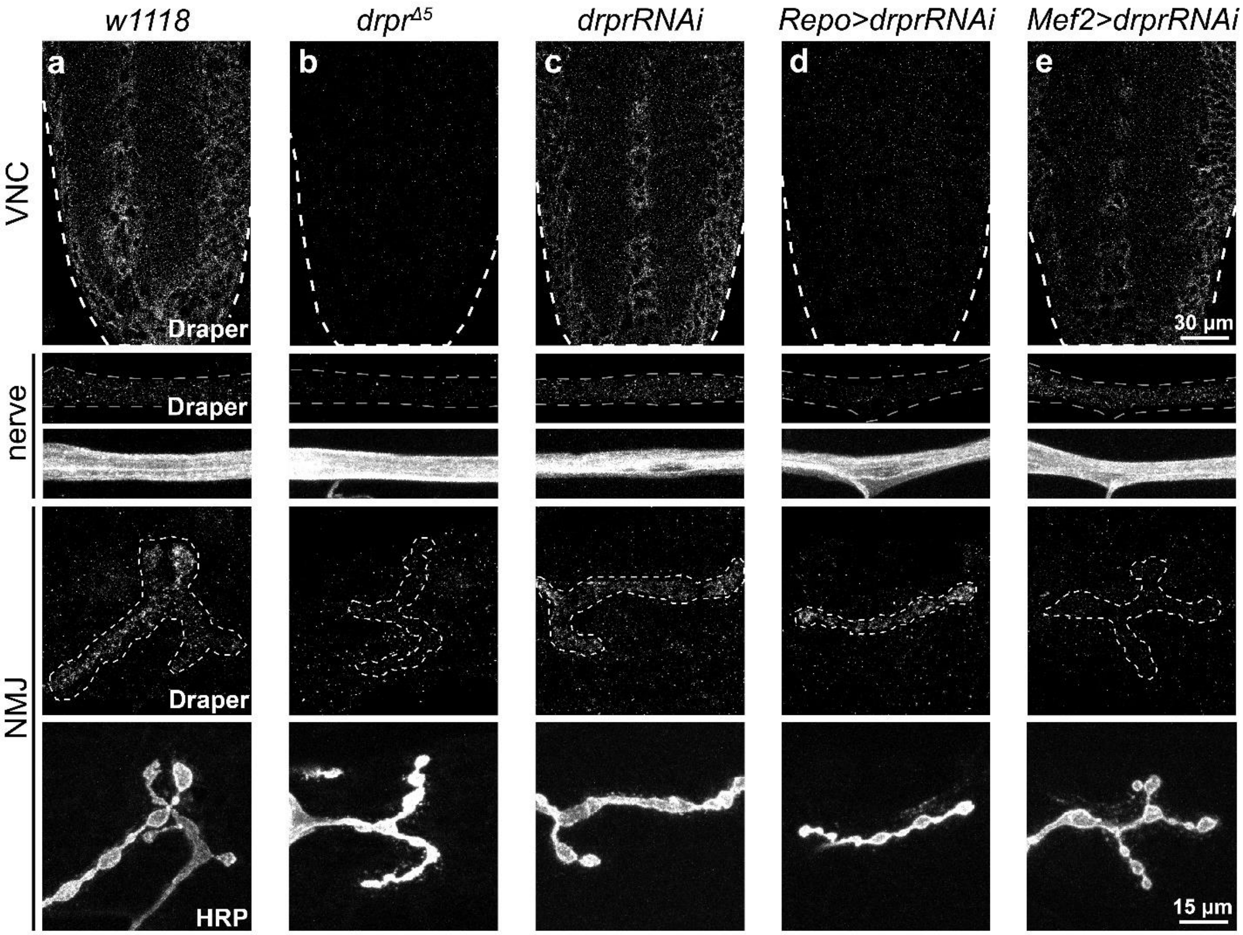

Supplementary Fig. 3. Validating *draper* RNAi efficiency.

VNC, segmental nerves, and NMJs of (a) *w1118*, (b) *draper* mutant (*drpr*$^{\Delta5}$), (c) *drprRNAi*, (d) glia *draper* knockdown (*Repo>drprRNAi*), and (e) muscle *draper* knockdown (*Mef2>drprRNAi*), stained with Draper and HRP. Note that glia *draper* knockdown completely removed the Draper signal from the VNC and segmental nerve, and muscle *draper* knockdown completely removed the Draper signal at the NMJs. 6 larvae were examined from each genotype.

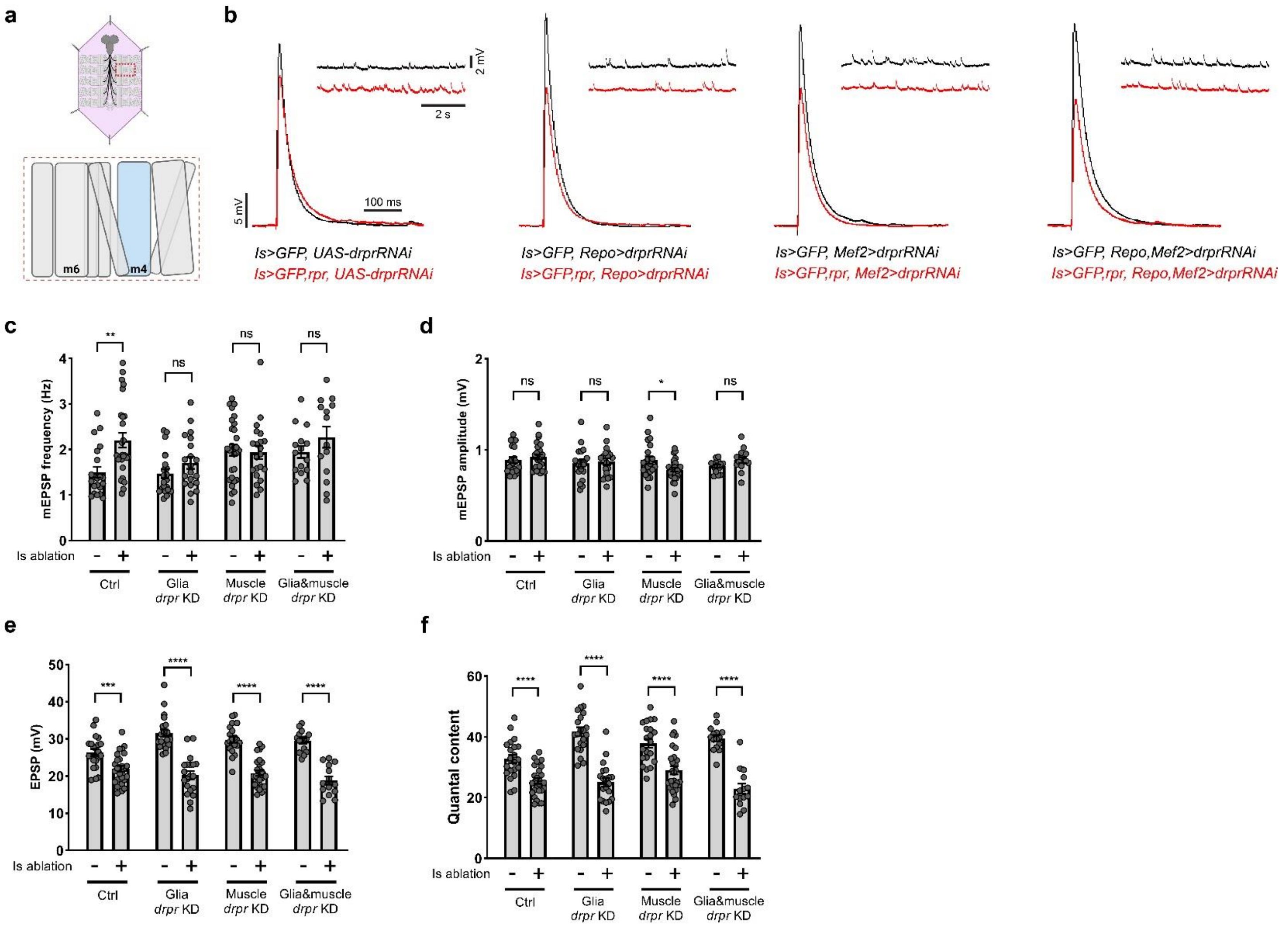

Supplementary Fig. 4. Electrophysiology recordings in cell type specific *draper* knockdown larvae.

a. Cartoon representation of a dissected larva (pink) and a hemisegment highlighted by dashed red rectangle. The target muscle examined in this figure is shown in blue. Cartoon is generated with Biorender.

b. EPSP and mEPSP recordings of non-ablated and Is ablated larvae in control, glia *draper* knockdown, muscle *draper* knockdown, and double knockdown backgrounds.

c. Quantification of mEPSP frequency of non-ablated and Is ablated larvae in control, glia *draper* knockdown, muscle *draper* knockdown, and double knockdown backgrounds. Control, $t(45)=3.352$, $p=0.0016$, unpaired t-test, two-tailed. Glia *draper* knockdown, $t(39)=1.429$, $p=0.1610$, unpaired t-test, two-tailed. Muscle *draper* knockdown, $t(47)=0.2739$, $p=0.7854$, unpaired t-test, two-tailed. Double knockdown, $t(27)=1.219$, $p=0.2333$, unpaired t-test, two-tailed.

d. Quantification of mEPSP amplitude of non-ablated and Is ablated larvae in control, glia *draper* knockdown, muscle *draper* knockdown, and double knockdown backgrounds. Control, $t(45)=0.8366$, $p=0.4072$, unpaired t-test, two-tailed. Glia *draper* knockdown, $t(39)=0.3657$, $p=0.7165$, unpaired t-test, two-tailed. Muscle *draper* knockdown, $t(29.93)=2.279$, $p=0.0300$, unpaired t-test, two-tailed, with Welch’s correction. Double knockdown, $t(20.18)=1.848$, $p=0.0793$, unpaired t-test Welch’s correction.

e. Quantification of EPSP amplitude of non-ablated and Is ablated larvae in control, glia *draper* knockdown, muscle *draper* knockdown, and double knockdown backgrounds. Control, $t(45)=3.579$, $p=0.0008$, unpaired t-test, two-tailed. Glia *draper* knockdown, $t(39)=7.543$, $p<0.0001$, unpaired t-test, two-tailed. Muscle *draper*

knockdown, t(47)=8.289, p<0.0001, unpaired t-test, two-tailed. Double knockdown, t(27)=8.247, p<0.0001, unpaired t-test, two-tailed.

f. Quantification of quantal content of non-ablated and Is ablated larvae in control, glia *draper* knockdown, muscle *draper* knockdown, and double knockdown backgrounds. Control, t(45)=4.472, p<0.0001, unpaired t-test, two-tailed. Glia *draper* knockdown, t(39)=8.157, p<0.0001, unpaired t-test, two-tailed. Muscle *draper* knockdown, t(47)=4.407, p<0.0001, unpaired t-test, two-tailed. Double knockdown, t(27)=8.579, p<0.0001, unpaired t-test, two-tailed. For c-f, N (NMJs) = 21, 26, 21, 20, 21, 28, 15, 14.

Error bars indicate ± SEM, ns = non-significant, *p<0.05, **p<0.01, ***p<0.001, ****p<0.0001.

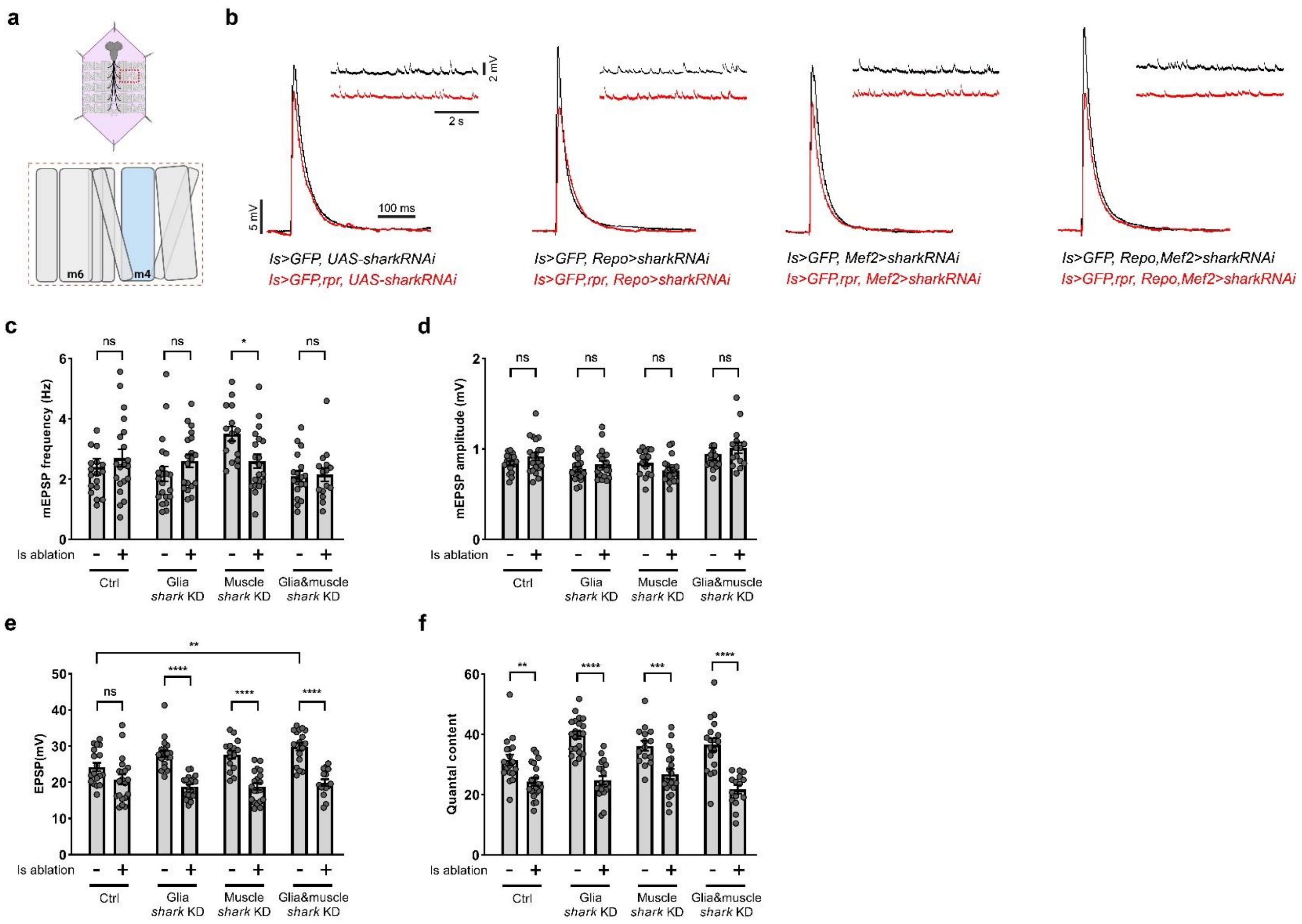

Supplementary Fig. 5. Electrophysiology recordings in cell type specific *shark* knock down larvae.

a. Cartoon representation of a dissected larva (pink) and a hemisegment highlighted by dashed red rectangle. The target muscle examined in this figure is shown in blue. Cartoon is generated with Biorender.

b. EPSP and mEPSP recordings of non-ablated and Is ablated larvae in control, glia *shark* knockdown, muscle *shark* knockdown and double knockdown backgrounds.

c. Quantification of mEPSP frequency of non-ablated and Is ablated larvae in control, glia *shark* knockdown, muscle *shark* knockdown, and double knockdown backgrounds. Control, t(36)=0.7421, p=0.4629, unpaired t-test, two-tailed. Glia *shark* knockdown, t(39)=1.300, p=0.2015, unpaired t-test, two-tailed. Muscle *shark* knockdown, t(33)=2.710, p=0.0106, unpaired t-test, two-tailed. Double knockdown, t(31)=0.1874, p=0.8525, unpaired t-test, two-tailed.

d. Quantification of mEPSP amplitude of non-ablated and Is ablated larvae in control, glia *shark* knockdown, muscle *shark* knockdown, and double knockdown backgrounds. Control, t(29.11)=1.575, p=0.4629, unpaired t-test, two-tailed, with Welch's correction. Glia *shark* knockdown, t(39)=1.261, p=0.2149, unpaired t-test, two-tailed. Muscle *shark* knockdown, t(33)=1.748, p=0.0898, unpaired t-test, two-tailed. Double knockdown, t(31)=0.7594, p=0.4533, unpaired t-test, two-tailed.

e. Quantification of EPSP amplitude of non-ablated and Is ablated larvae in control, glia *shark* knockdown, muscle *shark* knockdown, and double knockdown backgrounds. Control, t(36)=1.889, p=0.0669, unpaired t-test, two-tailed. Glia *shark* knockdown, t(39)=8.230, p<0.0001, unpaired t-test, two-tailed. Muscle *shark* knockdown, t(33)=6.017, p<0.0001, unpaired t-test, two-tailed. Double knockdown, t(31)=6.823, p<0.0001, unpaired t-test, two-tailed.

f. Quantification of quantal content of non-ablated and Is ablated larvae in control, glia *shark* knockdown, muscle *shark* knockdown, and double knockdown backgrounds. Control, $t(36)=3.328$, $p=0.0020$, unpaired t-test, two-tailed. Glia *shark* knock down, $t(39)=8.159$, $p<0.0001$, unpaired t-test, two-tailed. Muscle *shark* knock down, $t(33)=3.878$, $p=0.0005$, unpaired t-test, two-tailed. Double knock down, $t(31)=5.581$, $p<0.0001$, unpaired t-test, two-tailed. For c-f, N (NMJs) = 18, 20, 21, 20, 15, 20, 18, 15.

Error bars indicate ± SEM, ns = non-significant, *p<0.05, **p<0.01, ***p<0.001, ****p<0.0001.

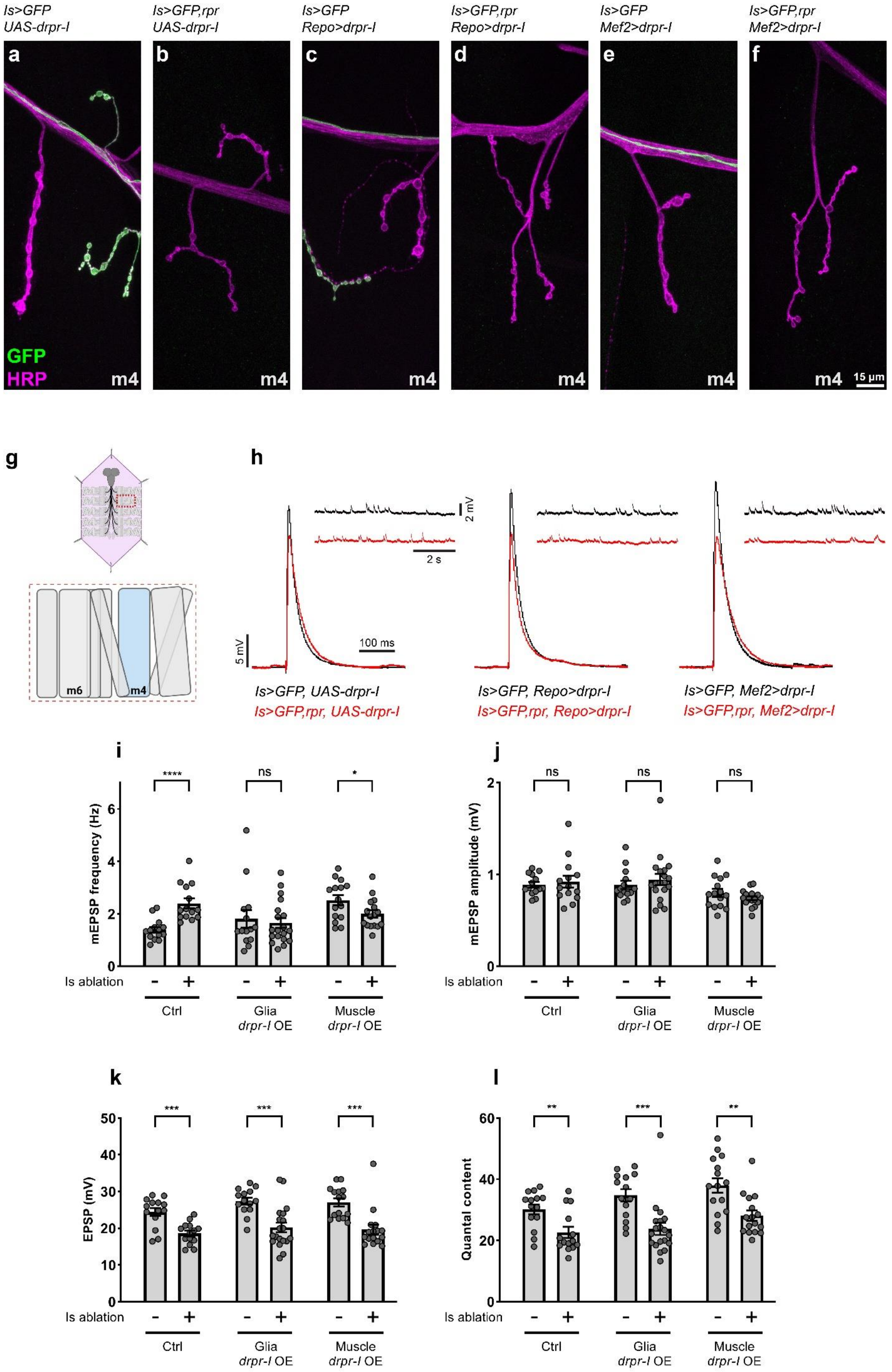

Is>GFP UAS-drpr-I
Is>GFP,rpr UAS-drpr-I
Is>GFP Repo>drpr-I
Is>GFP,rpr Repo>drpr-I
Is>GFP Mef2>drpr-I
Is>GFP,rpr Mef2>drpr-I
a
b
c
d
e
f
GFP
HRP
m4
15 µm
g
m6
m4
h
2 mV
2 s
5 mV
100 ms
Is>GFP, UAS-drpr-I
Is>GFP,rpr, UAS-drpr-I
Is>GFP, Repo>drpr-I
Is>GFP,rpr, Repo>drpr-I
Is>GFP, Mef2>drpr-I
Is>GFP,rpr, Mef2>drpr-I
i
mEPSP frequency (Hz)
****
ns
*
Is ablation
Ctrl
Glia drpr-I OE
Muscle drpr-I OE
j
mEPSP amplitude (mV)
ns
ns
ns
k
EPSP (mV)
***
***
***
l
Quantal content
**
***
**

Supplementary Fig. 6. Morphology and physiology of MN4-Ib in *draper-I* overexpressing larvae.

a-f. NMJs of MN4-Ib in third instar larvae of non-ablated and Is ablated larvae in control, glia *draper-I* overexpression, and muscle *draper-I* overexpression backgrounds, labeled with GFP (green) and HRP (magenta). Quantification is shown in Fig. 5b. N (NMJs) = 24, 23, 18, 23, 23 and 18.

g. Cartoon representation of a dissected larva (pink) and a hemisegment highlighted by dashed red rectangle. The target muscle examined in this figure is shown in blue. Cartoon is generated with Biorender.

h. EPSP and mEPSP recordings from larvae of displayed genotypes.

i. Quantification of mEPSP frequency of non-ablated and Is ablated larvae in control, glia *draper-I* overexpression, and muscle *draper-I* overexpression backgrounds. Control, $t(26)=4.689$, $p<0.0001$, unpaired t-test, two-tailed. Glia *draper-I* overexpression, $t(31)=0.4401$, $p=0.6629$, unpaired t-test, two-tailed. Muscle *draper-I* overexpression, $t(29)=2.193$, $p=0.0365$, unpaired t-test, two-tailed.

j. Quantification of mEPSP amplitude of non-ablated and Is ablated larvae in control, glia *draper-I* overexpression, and muscle *draper-I* overexpression backgrounds. Control, $t(18.40)=0.4746$, $p=0.6407$, unpaired t-test, two-tailed, with Welch's correction. Glia *draper-I* overexpression, $t(31)=0.7314$, $p=0.4700$, unpaired t-test, two-tailed. Muscle *draper-I* overexpression, $t(21.29)=1.243$, $p=0.2274$, unpaired t-test, two-tailed, with Welch's correction.

k. Quantification of EPSP amplitude of non-ablated and Is ablated larvae in control, glia *draper-I* overexpression, and muscle *draper-I* overexpression backgrounds. Control, $t(26)=4.322$, $p=0.0002$, unpaired t-test, two-tailed. Glia *draper-I* overexpression, $t(31)=4.073$, $p=0.0003$, unpaired t-test, two-tailed. Muscle *draper-I* overexpression, $t(29)=4.251$, $p=0.0002$, unpaired t-test, two-tailed.

l. Quantification of quantal content of non-ablated and Is ablated larvae in control, glia *draper-I* overexpression, and muscle *draper-I* overexpression backgrounds. Control, $t(26)=3.076$, $p=0.0049$, unpaired t-test, two-tailed. Glia *draper-I* overexpression, $t(31)=3.802$, $p=0.0006$, unpaired t-test, two-tailed. Muscle *draper-I* overexpression, $t(29)=3.440$, $p=0.0018$, unpaired t-test, two-tailed. For i-l, N (NMJs) = 14, 14, 14, 19, 15, 16.

Error bars indicate ± SEM, ns = non-significant, *$p<0.05$, **$p<0.01$, ***$p<0.001$, ****$p<0.0001$.

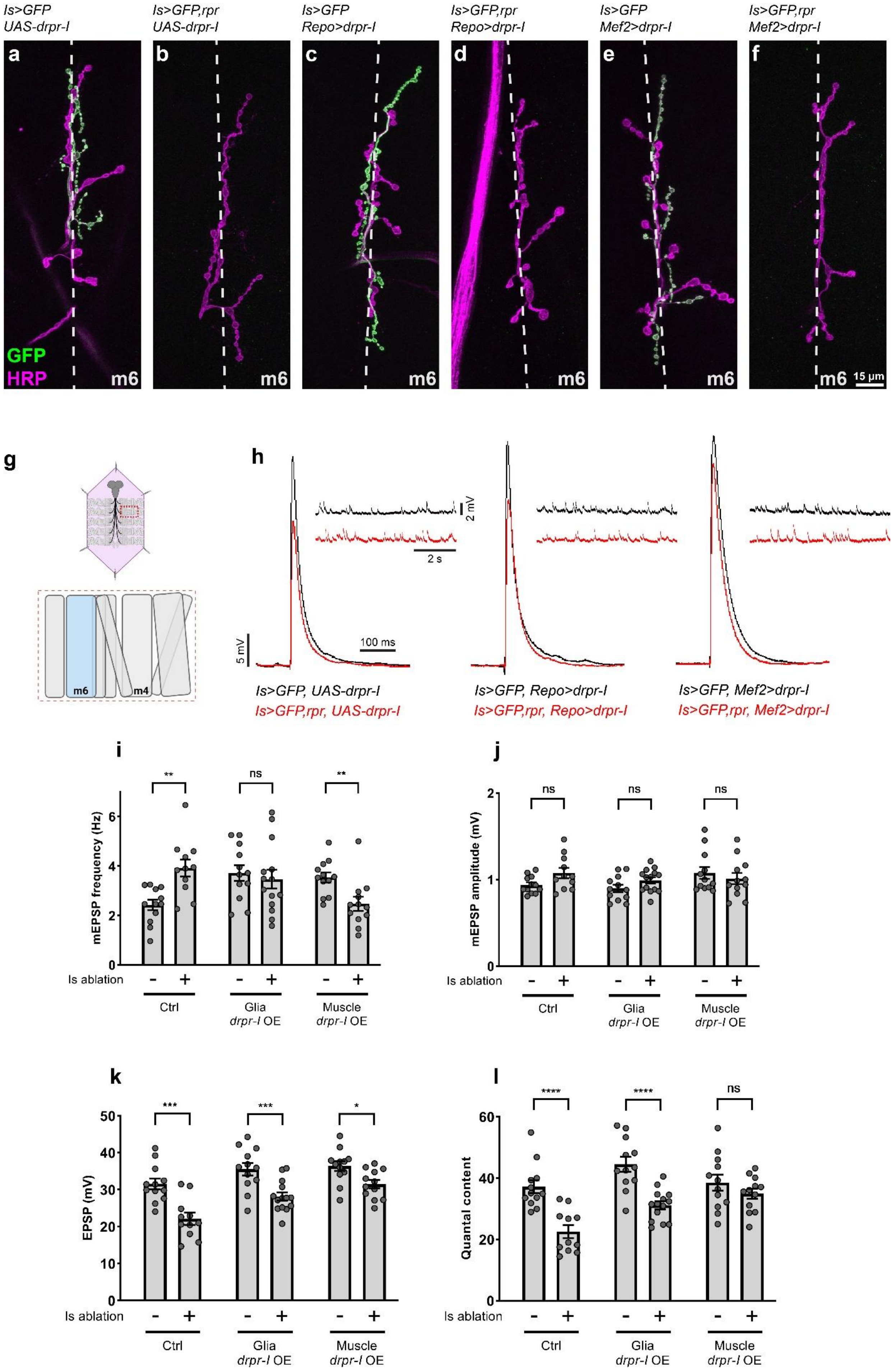

Is>GFP
UAS-drpr-I
Is>GFP,rpr
UAS-drpr-I
Is>GFP
Repo>drpr-I
Is>GFP,rpr
Repo>drpr-I
Is>GFP
Mef2>drpr-I
Is>GFP,rpr
Mef2>drpr-I
a
b
c
d
e
f
GFP
HRP
m6
15 µm
g
m6
m4
h
2 mV
2 s
100 ms
5 mV
Is>GFP, UAS-drpr-I
Is>GFP,rpr, UAS-drpr-I
Is>GFP, Repo>drpr-I
Is>GFP,rpr, Repo>drpr-I
Is>GFP, Mef2>drpr-I
Is>GFP,rpr, Mef2>drpr-I
i
mEPSP frequency (Hz)
** ns **
Is ablation - + - + - +
Ctrl
Glia drpr-I OE
Muscle drpr-I OE
j
mEPSP amplitude (mV)
ns ns ns
k
EPSP (mV)
*** *** *
l
Quantal content
**** **** ns

Supplementary Fig. 7. Morphology and physiology of MN6-Ib in *draper-I* overexpressing larvae.

a-f. NMJs of MN6-Ib in third instar larvae of non-ablated and Is ablated larvae in control, glia *draper-I* overexpression, and muscle *draper-I* overexpression backgrounds, labeled with GFP (green) and HRP (magenta). Quantification is shown in Fig. 5f. N (NMJs) = 19, 23, 20, 18, 22 and 16.

g. Cartoon representation of a dissected larva (pink) and a hemisegment highlighted by dashed red rectangle. The target muscle examined in this figure is shown in blue. Cartoon is generated with Biorender.

h. EPSP and mEPSP recordings from larvae of displayed genotypes.

i. Quantification of mEPSP frequency of non-ablated and Is ablated larvae in control, glia *draper-I* overexpression, and muscle *draper-I* overexpression backgrounds. Control, $t(21)=3.734$, $p=0.0012$, unpaired t-test, two-tailed. Glia *draper-I* overexpression, $t(24)=0.4865$, $p=0.6310$, unpaired t-test, two-tailed. Muscle *draper-I* overexpression, $t(22)=3.059$, $p=0.0057$, unpaired t-test, two-tailed.

j. Quantification of mEPSP amplitude of non-ablated and Is ablated larvae in control, glia *draper-I* overexpression, and muscle *draper-I* overexpression backgrounds. Control, $t(14.48)=2.050$, $p=0.0589$, unpaired t-test, two-tailed, with Welch's correction. Glia *draper-I* overexpression, $t(24)=1.707$, $p=0.1007$, unpaired t-test, two-tailed. Muscle *draper-I* overexpression, $t(22)=0.7120$, $p=0.4839$, unpaired t-test, two-tailed.

k. Quantification of EPSP amplitude of non-ablated and Is ablated larvae in control, glia *draper-I* overexpression, and muscle *draper-I* overexpression backgrounds. Control, $t(21)=4.324$, $p=0.0003$, unpaired t-test, two-tailed. Glia *draper-I* overexpression, $t(24)=3.757$, $p=0.0010$, unpaired t-test, two-tailed. Muscle *draper-I* overexpression, $t(22)=2.806$, $p=0.0103$, unpaired t-test, two-tailed.

l. Quantification of quantal content of non-ablated and Is ablated larvae in control, glia *draper-I* overexpression, and muscle *draper-I* overexpression backgrounds. Control, $t(21)=4.858$, $p<0.0001$, unpaired t-test, two-tailed. Glia *draper-I* overexpression, $t(24)=5.024$, $p<0.0001$, unpaired t-test, two-tailed. Muscle *draper-I* overexpression, $t(22)=1.164$, $p=0.2569$, unpaired t-test, two-tailed. For i-l, N (NMJs) = 12, 11, 12, 14, 12, 12.

Error bars indicate ± SEM, ns = non-significant, *$p<0.05$, **$p<0.01$, ***$p<0.001$, ****$p<0.0001$.

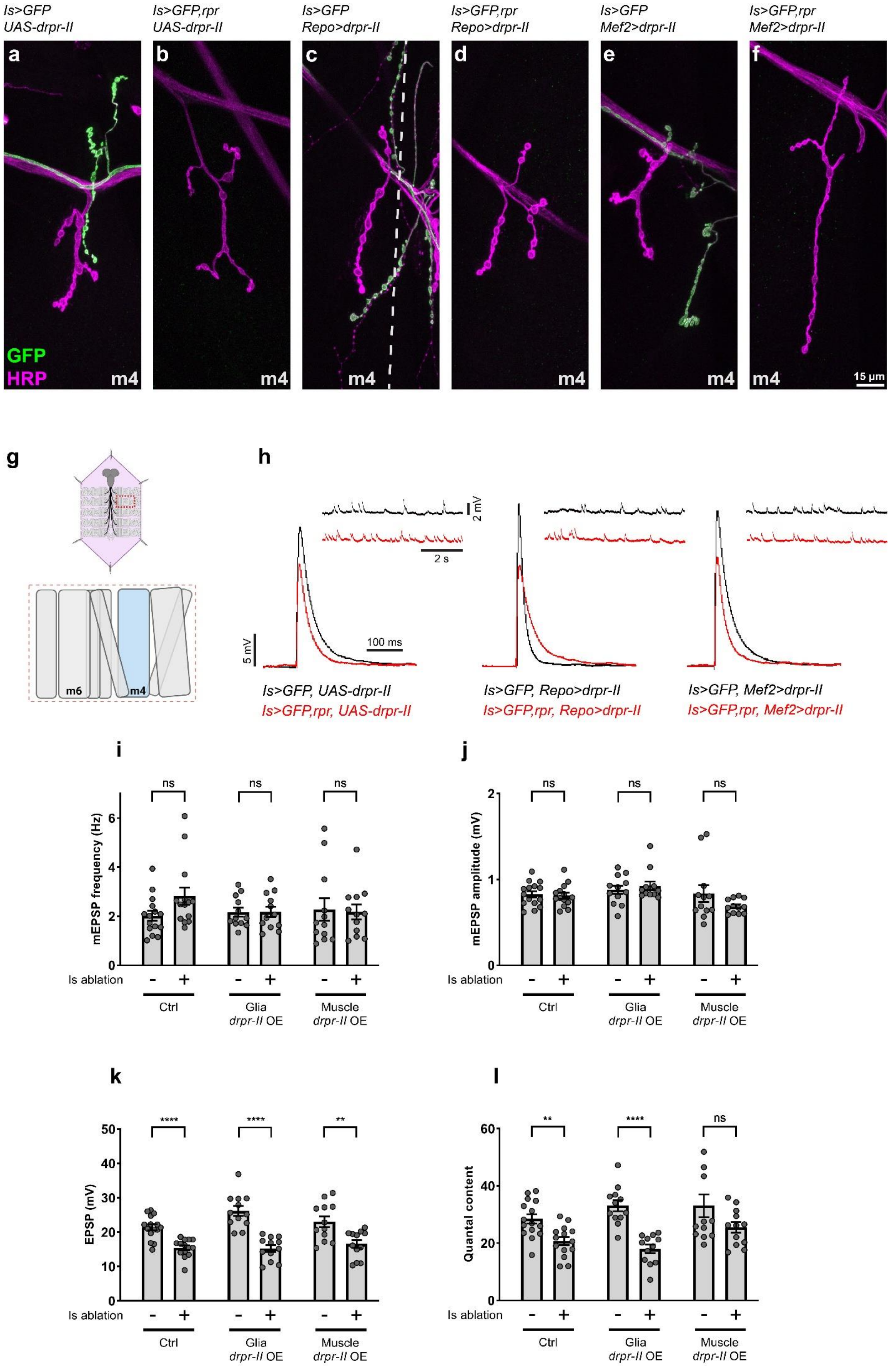

Is>GFP
UAS-drpr-II
Is>GFP,rpr
UAS-drpr-II
Is>GFP
Repo>drpr-II
Is>GFP,rpr
Repo>drpr-II
Is>GFP
Mef2>drpr-II
Is>GFP,rpr
Mef2>drpr-II
a
b
c
d
e
f
GFP
HRP
m4
15 µm
g
m6
m4
h
2 mV
2 s
5 mV
100 ms
Is>GFP, UAS-drpr-II
Is>GFP,rpr, UAS-drpr-II
Is>GFP, Repo>drpr-II
Is>GFP,rpr, Repo>drpr-II
Is>GFP, Mef2>drpr-II
Is>GFP,rpr, Mef2>drpr-II
i
mEPSP frequency (Hz)
ns
Is ablation
Ctrl
Glia
drpr-II OE
Muscle
drpr-II OE
j
mEPSP amplitude (mV)
k
EPSP (mV)
****
**
l
Quantal content

Supplementary Fig. 8. Morphology and physiology of MN4-Ib in *draper-II* overexpressing larvae.

a-f. NMJs of MN4-Ib in third instar larvae of non-ablated and Is ablated larvae in control, glia *draper-II* overexpression, and muscle *draper-II* overexpression background, labeled with GFP (green) and HRP (magenta). Quantification is shown in Fig. 6b. N (NMJs) = 19, 24, 19, 23, 18 and 23.

g. Cartoon representation of a dissected larva (pink) and a hemisegment highlighted by dashed red rectangle. The target muscle examined in this figure is shown in blue. Cartoon is generated with Biorender.

h. EPSP and mEPSP recordings from larvae of displayed genotypes.

i. Quantification of mEPSP frequency of non-ablated and Is ablated larvae in control, glia *draper-II* overexpression, and muscle *draper-II* overexpression backgrounds. Control, t(27)=1.990, p=0.0568, unpaired t-test, two-tailed. Glia *draper-II* overexpression, t(22)=0.040, p=0.9684, unpaired t-test, two-tailed. Muscle *draper-II* overexpression, t(22)=0.1858, p=0.8543, unpaired t-test, two-tailed.

j. Quantification of mEPSP amplitude of non-ablated and Is ablated larvae in control, glia *draper-II* overexpression, and muscle *draper-II* overexpression backgrounds. Control, t(27.00)=0.3215, p=0.7503, unpaired t-test, two-tailed, with Welch's correction. Glia *draper-II* overexpression, t(22)=0.6748, p=0.5068, unpaired t-test, two-tailed. Muscle *draper-II* overexpression, t(12.32)=1.455, p=0.1706, unpaired t-test, two-tailed, with Welch's correction.

k. Quantification of EPSP amplitude of non-ablated and Is ablated larvae in control, glia *draper-II* overexpression, and muscle *draper-II* overexpression backgrounds. Control, t(27)=5.214, p<0.0001, unpaired t-test, two-tailed. Glia *draper-II* overexpression, t(22)=6.311, p<0.0001, unpaired t-test, two-tailed. Muscle *draper-II* overexpression, t(22)=3.319, p=0.0031, unpaired t-test, two-tailed.

l. Quantification of quantal content of non-ablated and Is ablated larvae in control, glia *draper-II* overexpression, and muscle *draper-II* overexpression background. Control, t(27)=3.496, p=0.0017, unpaired t-test, two-tailed. Glia *draper-II* overexpression, t(22)=6.236, p<0.0001, unpaired t-test, two-tailed. Muscle *draper-II* overexpression, t(15.48)=1.715, p=0.1063, unpaired t-test, two-tailed, with Welch's correction. For i-l, N (NMJs) = 15, 14, 12, 12, 12, 12.

Error bars indicate ± SEM, ns = non-significant, **p<0.01, ****p<0.0001.

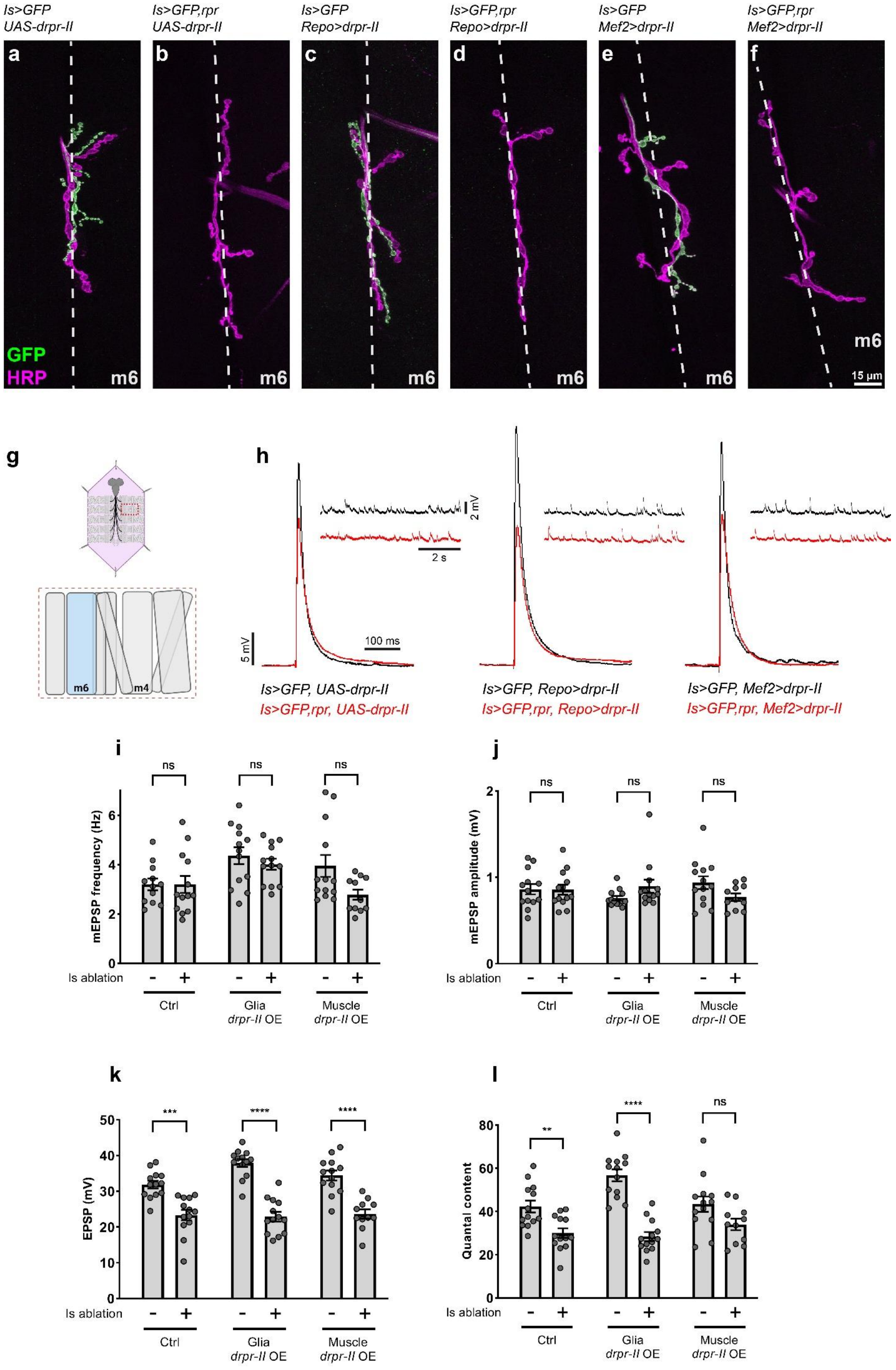
Is>GFP UAS-drpr-II
Is>GFP,rpr UAS-drpr-II
Is>GFP Repo>drpr-II
Is>GFP,rpr Repo>drpr-II
Is>GFP Mef2>drpr-II
Is>GFP,rpr Mef2>drpr-II
a
b
c
d
e
f
GFP
HRP
m6
15 µm
g
m6
m4
h
2 mV
2 s
5 mV
100 ms
Is>GFP, UAS-drpr-II
Is>GFP,rpr, UAS-drpr-II
Is>GFP, Repo>drpr-II
Is>GFP,rpr, Repo>drpr-II
Is>GFP, Mef2>drpr-II
Is>GFP,rpr, Mef2>drpr-II
i
mEPSP frequency (Hz)
ns
Is ablation
Ctrl
Glia drpr-II OE
Muscle drpr-II OE
j
mEPSP amplitude (mV)
k
EPSP (mV)
***
****
l
Quantal content
**

Supplementary Fig. 9. Morphology and physiology of MN6-Ib in *draper-II* overexpressing larvae.

a-f. NMJs of MN6-Ib in third instar larvae of non-ablated and Is ablated larvae in control, glia *draper-II* overexpression, and muscle *draper-II* overexpression background, labeled with GFP (green) and HRP (magenta). Quantification is shown in Fig. 6f. N (NMJs) = 19, 19, 23, 17, 16 and 20.

g. Cartoon representation of a dissected larva (pink) and a hemisegment highlighted by dashed red rectangle. The target muscle examined in this figure is shown in blue. Cartoon is generated with Biorender.

h. EPSP and mEPSP recordings from larvae of displayed genotypes.

i. Quantification of mEPSP frequency of non-ablated and Is ablated larvae in control, glia *draper-II* overexpression, and muscle *draper-II* overexpression backgrounds. Control, t(24)=0.0086, p=0.9932, unpaired t-test, two-tailed. Glia *draper-II* overexpression, t(24)=0.8540, p=0.4015, unpaired t-test, two-tailed. Muscle *draper-II* overexpression, t(22)=2.237, p=0.0358, unpaired t-test, two-tailed.

j. Quantification of mEPSP amplitude of non-ablated and Is ablated larvae in control, glia *draper-II* overexpression, and muscle *draper-II* overexpression backgrounds. Control, t(24)=0.0733, p=0.9422, unpaired t-test, two-tailed. Glia *draper-II* overexpression, t(24)=1.733, p=0.0959, unpaired t-test, two-tailed. Muscle *draper-II* overexpression, t(22)=1.841, p=0.0791, unpaired t-test, two-tailed.

k. Quantification of EPSP amplitude of non-ablated and Is ablated larvae in control, glia *draper-II* overexpression, and muscle *draper-II* overexpression backgrounds. Control, t(24)=4.647, p=0.0001, unpaired t-test, two-tailed. Glia *draper-II* overexpression, t(24)=8.613, p<0.0001, unpaired t-test, two-tailed. Muscle *draper-II* overexpression, t(22)=5.633, p<0.0001, unpaired t-test, two-tailed.

l. Quantification of quantal content of non-ablated and Is ablated larvae in control, glia *draper-II* overexpression, and muscle *draper-II* overexpression background. Control, t(24)=3.466, p=0.0020, unpaired t-test, two-tailed. Glia *draper-II* overexpression, t(24)=8.142, p<0.0001, unpaired t-test, two-tailed. Muscle *draper-II* overexpression, t(22)=2.044, p=0.0531, unpaired t-test, two-tailed. For i-l, N (NMJs) = 13, 13, 13, 13, 13, 11.

Error bars indicate ± SEM, ns = non-significant, **p<0.01, ***p<0.001, ****p<0.0001.

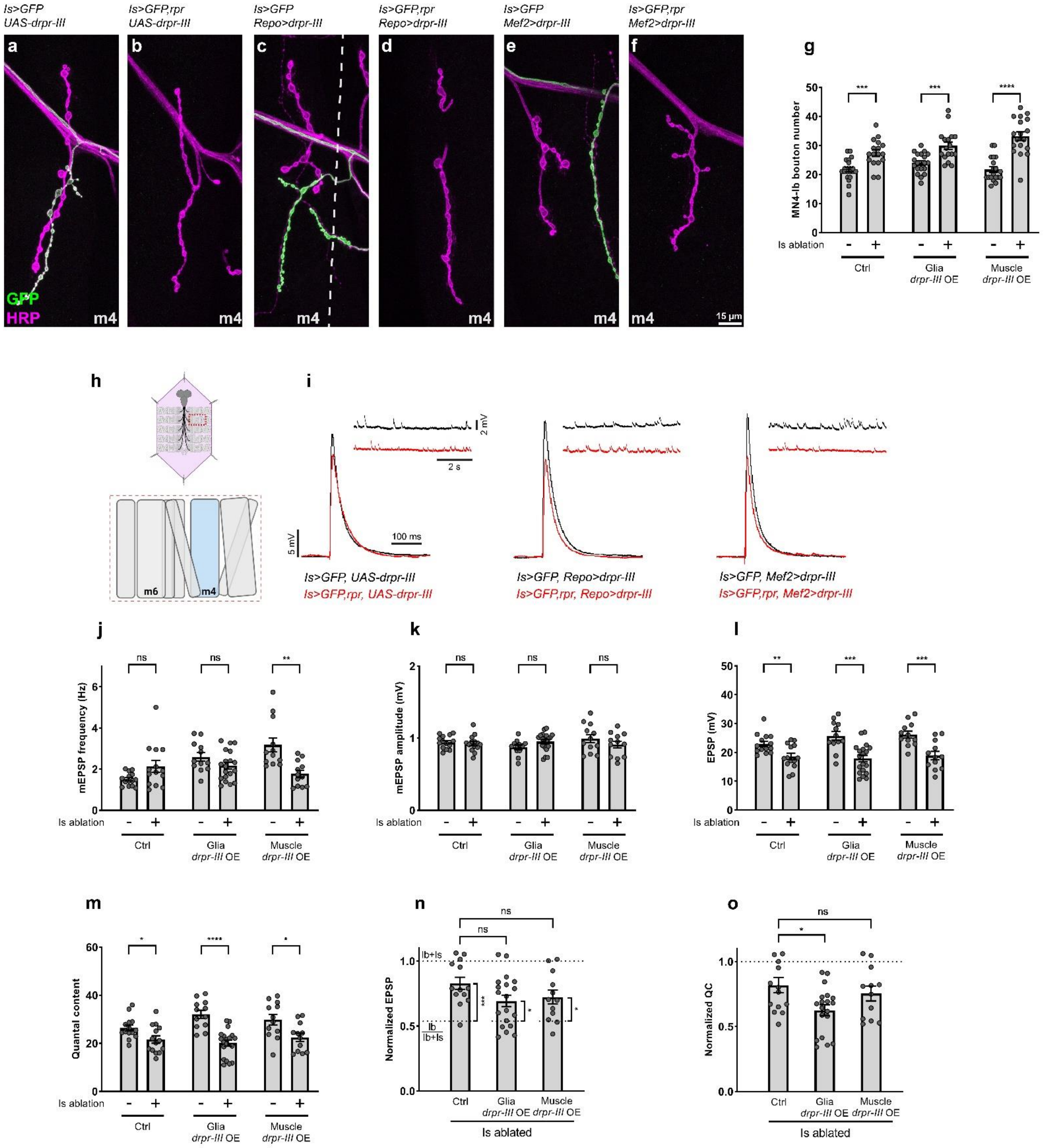

Supplementary Fig. 10. Morphology and physiology of MN4-Ib in *draper-III* overexpressing larvae.

a-f. NMJs of MN4-Ib in third instar larvae of non-ablated and Is ablated larvae in control, glia *draper-III* overexpression, and muscle *draper-III* overexpression backgrounds, labeled with GFP (green) and HRP (magenta). Quantification is shown in g. N (NMJs) = 21, 17, 19, 17, 19 and 18.

g. Quantification of MN4-Ib bouton number in non-ablated and Is ablated larvae in control, glia *draper-III* overexpression, and muscle *draper-III* overexpression backgrounds. Control (N = 21 and 17 NMJs), t(36)=4.232, p=0.0002 unpaired t-test, two-tailed. Glia *draper-III* overexpression (N = 19 and 17 NMJs), t(34)=3.961, p=0.0004, unpaired t-test, two-tailed. Muscle *draper-III* overexpression (N = 19 and 18 NMJs), t(35)=6.529, p<0.0001, unpaired t-test, two-tailed.

h. Cartoon representation of a dissected larva (pink) and a hemisegment highlighted by dashed red rectangle. The target muscle examined in this figure is shown in blue. Cartoon is generated with Biorender.

i. EPSP and mEPSP recordings from larvae of displayed genotypes.

j. Quantification of mEPSP frequency of non-ablated and Is ablated larvae in control, glia *draper-III* overexpression, and muscle *draper-III* overexpression backgrounds. Control, $t(15.25)=2.075$, $p=0.0553$, unpaired t-test, two-tailed, with Welch's correction. Glia *draper-III* overexpression, $t(29)=1.595$, $p=0.1216$, unpaired t-test, two-tailed. Muscle *draper-III* overexpression, $t(16.40)=3.626$, $p=0.0022$, unpaired t-test, two-tailed, with Welch's correction.

k. Quantification of mEPSP amplitude of non-ablated and Is ablated larvae in control, glia *draper-III* overexpression, and muscle *draper-III* overexpression backgrounds. Control, $t(26)=0.4944$, $p=0.6252$, unpaired t-test, two-tailed. Glia *draper-III* overexpression, $t(29)=1.781$, $p=0.0853$, unpaired t-test, two-tailed. Muscle *draper-III* overexpression, $t(22)=1.119$, $p=0.2753$, unpaired t-test, two-tailed.

l. Quantification of EPSP amplitude of non-ablated and Is ablated larvae in control, glia *draper-III* overexpression, and muscle *draper-III* overexpression backgrounds. Control, $t(26)=3.060$, $p=0.0051$, unpaired t-test, two-tailed. Glia *draper-III* overexpression, $t(29)=4.176$, $p=0.0002$, unpaired t-test, two-tailed. Muscle *draper-III* overexpression, $t(22)=3.939$, $p=0.0007$, unpaired t-test, two-tailed.

m. Quantification of quantal content of non-ablated and Is ablated larvae in control, glia *draper-III* overexpression, and muscle *draper-III* overexpression backgrounds. Control, $t(26)=2.452$, $p=0.0212$, unpaired t-test, two-tailed. Glia *draper-III* overexpression, $t(29)=5.630$, $p<0.0001$, unpaired t-test, two-tailed. Muscle *draper-III* overexpression, $t(22)=2.646$, $p=0.0147$, unpaired t-test, two-tailed. For j-m, N (NMJs) = 14, 14, 12, 19, 12, 12.

n. Quantification of normalized EPSP of Is ablated larvae in control, glia *draper-III* overexpression, and muscle *draper-III* overexpression backgrounds. $F(2, 41)=0.4426$, $p= 0.6454$, one-way ANOVA. Is ablated control vs glia *draper-III* overexpression, $p=0.1191$. Is ablated control vs muscle *draper-III* overexpression, $p=0.3328$. Is ablated control vs Ib/Ib+Is, $t(23)=4.613$, $p=0.0001$, unpaired t-test, two-tailed. Is ablated in glia *draper-III* overexpression vs Ib/Ib+Is, $t(29)=2.088$, $p=0.0457$, unpaired t-test, two-tailed. Is ablated in muscle *draper-III* overexpression vs Ib/Ib+Is, $t(22)=2.530$, $p=0.0191$, unpaired t-test, two-tailed.

o. Quantification of normalized quantal content of Is ablated larvae in control, glia *draper-III* overexpression, and muscle *draper-III* overexpression backgrounds. $F(2, 42)=0.4639$, $p=0.6320$, one-way ANOVA. Is ablated control vs glia *draper-III* overexpression, $p=0.0219$. Is ablated control vs muscle *draper-III* overexpression, $p=0.6899$. For n and o, N (NMJs) = 14, 19, 12.

Error bars indicate ± SEM, ns = non-significant, *$p<0.05$, **$p<0.01$, ***$p<0.001$, ****$p<0.0001$.

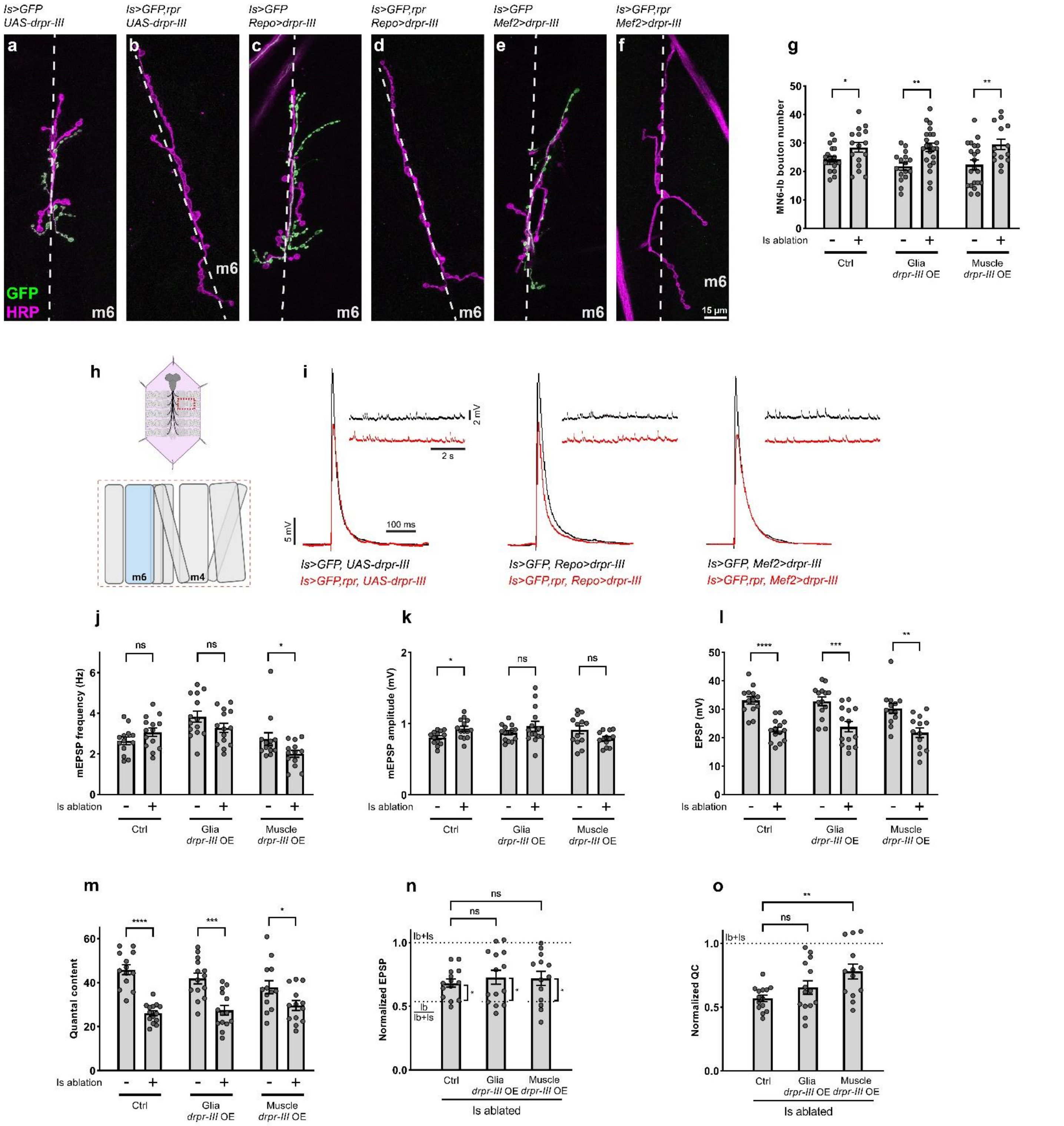

Supplementary Fig. 11. Morphology and physiology of MN6-Ib in *draper-III* overexpressing larvae.

a-f. NMJs of MN6-Ib in third instar larvae of non-ablated and Is ablated larvae in control, glia *draper-III* overexpression, and muscle *draper-III* overexpression backgrounds, labeled with GFP (green) and HRP (magenta). Quantification is shown in g. N (NMJs) = 20, 16, 16, 22, 21 and 14.

g. Quantification of MN6-Ib bouton number of non-ablated and Is ablated larvae in control, glia *draper-III* overexpression, and muscle *draper-III* overexpression backgrounds. Control (N = 20 and 16 NMJs), t(23.64)=2.129, p=0.0439, unpaired t-test, two-tailed, with Welch's correction.. Glia *draper-III* overexpression (N = 16 and 22 NMJs), t(36)=3.261, p=0.0024, unpaired t-test, two-tailed. Muscle *draper-III* overexpression (N = 21 and 14 NMJs), t(33)=2.852, p=0.0074, unpaired t-test, two-tailed.

h. Cartoon representation of a dissected larva (pink) and a hemisegment highlighted by dashed red rectangle. The target muscle examined in this figure is shown in blue. Cartoon is generated with Biorender.

i. EPSP and mEPSP recordings from larvae of displayed genotypes.

j. Quantification of mEPSP frequency of non-ablated and Is ablated larvae in control, glia *draper-III* overexpression, and muscle *draper-III* overexpression backgrounds. Control, $t(25)=1.544$, $p=0.1350$, unpaired t-test, two-tailed. Glia *draper-III* overexpression, $t(26)=1.541$, $p=0.1354$, unpaired t-test, two-tailed. Muscle *draper-III* overexpression, $t(24)=2.079$, $p=0.0485$, unpaired t-test, two-tailed.

k. Quantification of mEPSP amplitude of non-ablated and Is ablated larvae in control, glia *draper-III* overexpression, and muscle *draper-III* overexpression backgrounds. Control, $t(25)=2.727$, $p=0.0115$, unpaired t-test, two-tailed. Glia *draper-III* overexpression, $t(26)=1.224$, $p=0.2319$, unpaired t-test, two-tailed. Muscle *draper-III* overexpression, $t(24)=1.943$, $p=0.0638$, unpaired t-test, two-tailed.

l. Quantification of EPSP amplitude of non-ablated and Is ablated larvae in control, glia *draper-III* overexpression, and muscle *draper-III* overexpression backgrounds. Control, $t(25)=6.298$, $p<0.0001$, unpaired t-test, two-tailed. Glia *draper-III* overexpression, $t(26)=3.814$, $p=0.0008$, unpaired t-test, two-tailed. Muscle *draper-III* overexpression, $t(24)=3.549$, $p=0.0016$, unpaired t-test, two-tailed.

m. Quantification of quantal content of non-ablated and Is ablated larvae in control, glia *draper-III* overexpression, and muscle *draper-III* overexpression backgrounds. Control, $t(25)=7.938$, $p<0.0001$, unpaired t-test, two-tailed. Glia *draper-III* overexpression, $t(26)=4.425$, $p=0.0002$, unpaired t-test, two-tailed. Muscle *draper-III* overexpression, $t(24)=2.208$, $p=0.0371$, unpaired t-test, two-tailed. For j-m, N (NMJs) = 13, 14, 14, 14, 13, 13.

n. Quantification of normalized EPSP of Is ablated larvae in control, glia *draper-III* overexpression, and muscle *draper-III* overexpression backgrounds. $F(2, 38)=0.2634$, $p= 0.7698$, one-way ANOVA. Is ablated control vs glia *draper-III* overexpression, $p=0.7729$. Is ablated control vs muscle *draper-III* overexpression, $p=0.8508$. Is ablated control vs Ib/Ib+Is, $t(22)=2.291$, $p=0.0319$, unpaired t-test, two-tailed. Is ablated in glia *draper-III* overexpression vs Ib/Ib+Is, $t(22)=2.299$, $p=0.0314$, unpaired t-test, two-tailed. Is ablated in muscle *draper-III* overexpression vs Ib/Ib+Is, $t(21)=2.214$, $p=0.0380$, unpaired t-test, two-tailed.

o. Quantification of normalized quantal content of Is ablated larvae in control, glia *draper-III* overexpression, and muscle *draper-III* overexpression backgrounds. $F(2, 38)=4.981$, $p=0.0120$, one-way ANOVA. Is ablated control vs glia *draper-III* overexpression, $p=0.4070$. Is ablated control vs muscle *draper-III* overexpression, $p=0.0089$. For n and o, N (NMJs) = 14, 14, 13.

Error bars indicate ± SEM, ns = non-significant, $^{*}p<0.05$, $^{**}p<0.01$, $^{***}p<0.001$, $^{****}p<0.0001$.

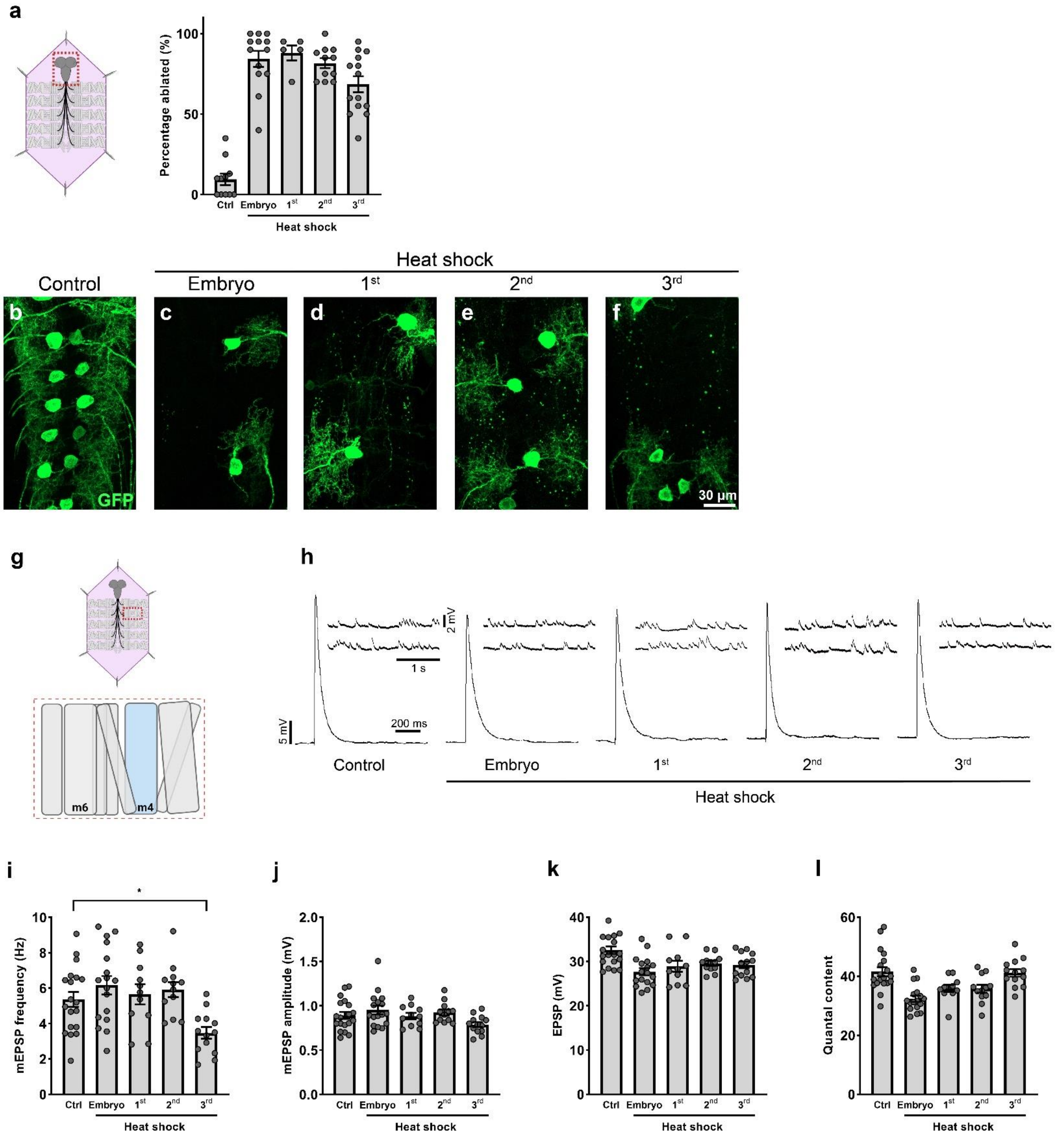

Supplementary Fig. 12. Morphology and electrophysiology of larvae with Is MNs ablated at different developmental stages.

a. Cartoon representation of the VNC, where the ablation efficiency is measured. The ablation efficiency is represented by the percentage of ablated Is MNs. N (VNCs) = 11, 13, 5, 11 and 14. Cartoon is generated with Biorender.

b-f. VNCs of late third instar larvae (*hs-FLP,UAS-GFP/+;UAS-FRT stop FRT-hid-2A-rpr/+;Is-GAL4/+*) with (b) no heat-shock, (c) embryo heat-shock, (d) first instar heat-shock, (e) second instar heat-shock, and (f) third

instar heat-shock, stained with GFP (green). Debris of the ablated Is MN cell body is fully removed in all conditions.

g. Cartoon representation of a dissected larva (pink) and a hemisegment highlighted by dashed red rectangle. The target muscle examined in this figure is shown in blue (i-l). Cartoon is generated with Biorender.

h. EPSP and mEPSP recordings from larvae with Is MNs ablated at different developmental stages.

i. Quantification of mEPSP frequency. $F(4, 67)=4.926$, $p=0.0015$. Control vs third instar heat-shocked, $p=0.0340$, One-way ANOVA.

j. Quantification of mEPSP amplitude. $F(4, 67)=2.248$, $p=0.0730$, One-way ANOVA.

k. Quantification of EPSP amplitude. $F(4, 67)=5.399$, $p=0.0008$, One-way ANOVA.

l. Quantification of quantal content. $F(4, 67)=9.109$, $p<0.0001$, One-way ANOVA. For i-l, N (NMJs) = 19, 17, 11, 12, 13.

Error bars indicate ± SEM, $*p<0.05$.
