# Supplementary material for: Glial Draper signaling triggers cross-neuron plasticity in bystander neurons after neuronal cell death in Drosophila
Source: Nat Commun. 2023 Jul 24;14:4452. doi: 10.1038/s41467-023-40142-y (PMC10366216; doi:10.1038/s41467-023-40142-y)
Supplement: Supplementary file 3 — Description of Additional Supplementary Files [file 41467_2023_40142_MOESM3_ESM.docx]

**Description of Additional Supplementary Files**

**Supplementary Movie 1:**

Representative movie of roll behavior of wild type and draper mutant larvae
