# Supplementary material for: Glial Draper signaling triggers cross-neuron plasticity in bystander neurons after neuronal cell death in Drosophila
Source: Nat Commun. 2023 Jul 24;14:4452. doi: 10.1038/s41467-023-40142-y (PMC10366216; doi:10.1038/s41467-023-40142-y)
Supplement: Supplementary file 5 — Reporting Summary [file 41467_2023_40142_MOESM5_ESM.pdf]

nature portfolio

Corresponding author(s): Carrillo, R.A. and Wang, Y.

Last updated by author(s): Jun 25, 2023

Reporting Summary

Nature Portfolio wishes to improve the reproducibility of the work that we publish. This form provides structure for consistency and transparency in reporting. For further information on Nature Portfolio policies, see our Editorial Policies and the Editorial Policy Checklist.

Statistics

For all statistical analyses, confirm that the following items are present in the figure legend, table legend, main text, or Methods section.

n/a	Confirmed	
☐	☒	The exact sample size (*n*) for each experimental group/condition, given as a discrete number and unit of measurement
☐	☒	A statement on whether measurements were taken from distinct samples or whether the same sample was measured repeatedly
☐	☒	The statistical test(s) used AND whether they are one- or two-sided<br>*Only common tests should be described solely by name; describe more complex techniques in the Methods section.*
☐	☒	A description of all covariates tested
☐	☒	A description of any assumptions or corrections, such as tests of normality and adjustment for multiple comparisons
☐	☒	A full description of the statistical parameters including central tendency (e.g. means) or other basic estimates (e.g. regression coefficient) AND variation (e.g. standard deviation) or associated estimates of uncertainty (e.g. confidence intervals)
☐	☒	For null hypothesis testing, the test statistic (e.g. *F*, *t*, *r*) with confidence intervals, effect sizes, degrees of freedom and *P* value noted<br>*Give P values as exact values whenever suitable.*
☒	☐	For Bayesian analysis, information on the choice of priors and Markov chain Monte Carlo settings
☒	☐	For hierarchical and complex designs, identification of the appropriate level for tests and full reporting of outcomes
☒	☐	Estimates of effect sizes (e.g. Cohen's *d*, Pearson's *r*), indicating how they were calculated

Our web collection on statistics for biologists contains articles on many of the points above.

Software and code

Policy information about availability of computer code

Data collection	Confocal images were acquired on a Zeiss LSM800 confocal microscope using either a 40X plan-neofluar 1.3 NA objective, or a 63X plan-apo 1.4 NA objective. Electrophysiology signals were amplified by a MultiClamp 700B amplifier (Molecular Devices), digitized with a Digidata 1550B (Molecular Devices), and acquired in pCLAMP 10 software (Molecular Devices). GCaMP signal is visualized under a Nikon FS microscope with a 40X long-working distance objective, and NMJ firing movies were recorded using PCO Edge 4.2 camera and NIS-Elements Imaging Software (Nikon, version 5.00). Rolling behavior movies were captured by an RPi camera (Waveshare) and Point Grey FlyCap2 software. Crawling trajectory was captured with a PiVR setup (Tadres et al., 2020, Plos Biology).
Data analysis	Confocal images are analyzed by ImageJ. Electrophysiology data is analyzed by Mini Analysis software (Synaptosoft). Crawling behavior is analyzed by customized code: https://github.com/sihaohuanguc/larva_trajectory_process. Statistical analysis is performed using Prism 9 (GraphPad).

For manuscripts utilizing custom algorithms or software that are central to the research but not yet described in published literature, software must be made available to editors and reviewers. We strongly encourage code deposition in a community repository (e.g. GitHub). See the Nature Portfolio guidelines for submitting code & software for further information.

Data

Policy information about availability of data

All manuscripts must include a data availability statement. This statement should provide the following information, where applicable:

- Accession codes, unique identifiers, or web links for publicly available datasets
- A description of any restrictions on data availability
- For clinical datasets or third party data, please ensure that the statement adheres to our policy

Original data is provided in Source Data file.

Human research participants

Policy information about studies involving human research participants and Sex and Gender in Research.

Reporting on sex and gender	This study did not include human samples.
Population characteristics	This study did not include human samples.
Recruitment	This study did not include human samples.
Ethics oversight	This study did not include human samples.

Note that full information on the approval of the study protocol must also be provided in the manuscript.

Field-specific reporting

Please select the one below that is the best fit for your research. If you are not sure, read the appropriate sections before making your selection.

☒ Life sciences ☐ Behavioural & social sciences ☐ Ecological, evolutionary & environmental sciences

For a reference copy of the document with all sections, see nature.com/documents/nr-reporting-summary-flat.pdf

Life sciences study design

All studies must disclose on these points even when the disclosure is negative.

Sample size	Sample sizes were determined by existing studies in the field to enable statistical analyses and reproducibility (Goel and Dickman, 2018, Nature Communications). In general, for each experiment, at least 10 total data points were collected from each genotype.
Data exclusions	No data were excluded.
Replication	All experiments were performed with at least two independent biological replicates and data points were pulled and analyzed together. All attempts at replication were successful.
Randomization	Male and female flies were collected and randomly assigned into different cross. Larvae were randomly selected for recording.
Blinding	Experimenters were blinded when perform bouton counting, and bouton numbers were linked with genotypes after counting. For other recordings, data is recorded by computer and no subjective rating of data is involved.

Reporting for specific materials, systems and methods

We require information from authors about some types of materials, experimental systems and methods used in many studies. Here, indicate whether each material, system or method listed is relevant to your study. If you are not sure if a list item applies to your research, read the appropriate section before selecting a response.

Materials & experimental systems

n/a	Involved in the study	
	x	Antibodies
x		Eukaryotic cell lines
x		Palaeontology and archaeology
	x	Animals and other organisms
x		Clinical data
x		Dual use research of concern

Methods

n/a	Involved in the study	
x		ChIP-seq
x		Flow cytometry
x		MRI-based neuroimaging

Antibodies

Antibodies used	Mouse anti-Repo, Developmental Studies Hybridoma Bank, #8D12, 1:50<br>Chicken anti-GFP, Michael Glotzer Lab, University of Chicago, 1:10,000<br>Rabbit anti-GFP, Thermo Fisher Scientific, #A11122, 1:500<br>Mouse anti-DLG, Developmental Studies Hybridoma Bank, #4F3, 1:100<br>Rabbit anti-DLG, Vivian Budnik Lab, UMass Chan Medical School (Koh et al., 1999), 1:40,000<br>Mouse anti-Draper, Developmental Studies Hybridoma Bank, #8A1-S, 1:30<br>Goat anti-HRP-Alexa Fluor 405, Jackson ImmunoResearch, #123-475-021, 1:100<br>Goat anti-HRP-TRITC, Jackson ImmunoResearch, #123-025-021, 1:100<br>Goat anti-HRP-Alexa Fluor 647, Jackson ImmunoResearch, #123-605-021, 1:100<br>Goat anti-mouse-Alexa Fluor 647, Thermo Fisher Scientific, #A32728, 1:500<br>Goat anti-chicken-Alexa Fluor 488, Thermo Fisher Scientific, #A11039, 1:500<br>Goat anti-rabbit-Alexa Fluor 568, Thermo Fisher Scientific, #A11036, 1:500<br>Goat anti-rabbit-Alexa Fluor 488, Thermo Fisher Scientific, #A11008, 1:500
Validation	Mouse anti-Repo, Developmental Studies Hybridoma Bank, #8D12 (Validation: https://dshb.biology.uiowa.edu/8D12-anti-Repo)<br>Chicken anti-GFP, Michael Glotzer Lab, University of Chicago<br>Rabbit anti-GFP, Thermo Fisher Scientific, #A11122 (Validation: https://www.thermofisher.com/antibody/product/GFP-Antibody-Polyclonal/A-11122)<br>Mouse anti-DLG, Developmental Studies Hybridoma Bank, #4F3 (Validation: https://dshb.biology.uiowa.edu/4F3-anti-discs-large)<br>Rabbit anti-DLG, Vivian Budnik Lab, UMass Chan Medical School (Koh et al., 1999)<br>Mouse anti-Draper, Developmental Studies Hybridoma Bank, #8A1-S (Validation: https://dshb.biology.uiowa.edu/Draper-8A1)<br>Goat anti-HRP-Alexa Fluor 405, Jackson ImmunoResearch, #123-475-021<br>Goat anti-HRP-TRITC, Jackson ImmunoResearch, #123-025-021<br>Goat anti-HRP-Alexa Fluor 647, Jackson ImmunoResearch, #123-605-021<br>Goat anti-mouse-Alexa Fluor 647, Thermo Fisher Scientific, #A32728<br>Goat anti-chicken-Alexa Fluor 488, Thermo Fisher Scientific, #A11039<br>Goat anti-rabbit-Alexa Fluor 568, Thermo Fisher Scientific, #A11036<br>Goat anti-rabbit-Alexa Fluor 488, Thermo Fisher Scientific, #A11008

Animals and other research organisms

Policy information about studies involving animals; ARRIVE guidelines recommended for reporting animal research, and Sex and Gender in Research

Laboratory animals	Adult Drosophila melanogaster 5 days after eclosion were used in this study. Stains involved are listed below with their sources:<br>w1118 (Carrillo et al., 2015)<br>A8-GAL4 (ls-GAL4), (Venkatasubramanian et al., 2019)<br>A8-LexA, Richard Mann<br>Repo-GAL4, Kai Zinn<br>Mef2-GAL4, Kai Zinn<br>10XUAS-mCD8::GFP, BL #32184<br>UAS-hid,rpr, (Zhou et al., 1997)<br>LexAOP-rpr, (Harris et al., 2020)<br>UAS-draper-RNAi, BL #67034<br>UAS-shark-RNAi, BL #42555<br>UAS-drprI, BL #67035<br>UAS-drprII, BL #67036<br>UAS-drprIII, BL #67037<br>MHC-CD8::GCaMP6f-sh, (Newman et al., 2017)<br>draperΔ5, (Freeman et al., 2003)<br>hs-FLP, BL #28832<br>UAS-FRT stop FRT-hid-2A-rpr, (Yu et al., 2020)

Wild animals	No wild animals were used in the study.
Reporting on sex	Both genders were equally used in this study.
Field-collected samples	This study did not include samples collected from the field.
Ethics oversight	No ethical approval is required for this study.

Note that full information on the approval of the study protocol must also be provided in the manuscript.
